# 9-cis retinoic acid induces neurorepair in stroke brain

**DOI:** 10.1038/s41598-017-04048-2

**Published:** 2017-07-03

**Authors:** Seong-Jin Yu, Mikko Airavaara, Kuo-Jen Wu, Brandon K Harvey, H. S. Liu, Yihong Yang, Alex Zacharek, Jieli Chen, Yun Wang

**Affiliations:** 1 Center for Neuropsychiatric Research, National Health Research Institutes, Zhunan, Taiwan; 2 Institute of Biotechnology, Viikki Biocenter, University of Helsinki, Helsinki, Finland; 3 Intramural Research Program, National Institute on Drug Abuse, NIH, USA; 4 Neurology, Henry Ford Hospital, Detroit, MI USA

## Abstract

The purpose of this study was to examine the neurorestorative effect of delayed 9 cis retinoic acid (9cRA) treatment for stroke. Adult male rats received a 90-min right distal middle cerebral artery occlusion (dMCAo). Animals were separated into two groups with similar infarction sizes, based on magnetic resonance imaging on day 2 after dMCAo. 9cRA or vehicle was given via an intranasal route daily starting from day 3. Stroke rats receiving 9cRA post-treatment showed an increase in brain 9cRA levels and greater recovery in motor function. 9cRA enhanced the proliferation of bromodeoxyuridine (+) cells in the subventricular zone (SVZ) and lesioned cortex in the stroke brain. Using subventricular neurosphere and matrigel cultures, we demonstrated that proliferation and migration of SVZ neuroprogenitor cells were enhanced by 9cRA. Our data support a delayed and non-invasive drug therapy for stroke. Intranasal 9cRA can facilitate the functional recovery and endogenous repair in the ischemic brain.

## Introduction

Stroke is the second leading global cause of death in the past decade (from the WHO, http://www.who.int/mediacentre/factsheets/fs310/en/index.html) and a leading cause of adult disability worldwide. Current clinical treatment strategies for stroke mainly focus on reduction of the dying cells early after the occurrence. This therapeutic approach, such as the use of thrombolytic agents for stroke, is limited by a narrow time window. No pharmacological agent has shown effectiveness in reducing the size of damage when therapy is initiated 3 days after stroke in patients^1^.

Cerebral ischemia can activate endogenous repair processes including *de novo* neurogenesis in the subventricular zone (SVZ) after stroke. The kinetic profile of neural progenitor cell (NPC) proliferation in the SVZ following ischemia has been examined using bromodeoxyuridine (BrdU) labeling^2^. A robust increase of BrdU immunoreactivity in the SVZ occurred as early as 2 days after distal middle cerebral artery occlusion (dMCAo). The increase of BrdU immunoreactivity was sustained through 4 days after dMCAo, started to decline between days 6 to 8, and returned to basal levels by day 10. In contrast, most of the cell death in the ischemic lesion area occurred by post-stroke day 2. These data suggest differential temporal windows of cell proliferation in the SVZ and cell death in the ischemic zone. Targeting the survival of the endogenous NPCs in SVZ may provide a long therapeutic window after onset of stroke^2, 3^.

Retinoic acid (RA) has been considered as a regeneration –inducing molecule, based on the reparative functions in the peripheral organs^4^. Two nuclear retinoic acid receptors, retinoic acid receptor (RAR) and retinoic X receptors (RXR), have been identified. 9-cis retinoic acid (9cRA) binds to the RXR with higher affinity and selectivity^5, 6^ than all-trans RA (atRA), an RAR agonist^7^. 9cRA has neurotrophic and neurodifferentiative properties. Exogenous 9cRA induced proliferation of immortalized hippocampal progenitor cells^8^, increased neurite outgrowth from the adult Lymmaea neurons in cell culture^9^, and accelerated remyelination in the injured CNS^10^. Knocking out RXR-γ inhibited the differentiation of cultured oligodendrocyte precursor cells^10^. The differentiation properties of 9cRA have also been found in neuroblastoma cells^11^. 9cRA is more potent than atRA in promoting neuronal differentiation of human neuroblastoma cells^12^. Taken together, these data suggest that 9cRA has neural trophic or reparative properties and may be potentially useful for post-stroke therapy.

Several studies have demonstrated that 9cRA has an indirect trophic action through other proteins. 9cRA upregulates the expression of bone morphogenetic protein 7 (BMP7) in rat neocortical culture^13^ and human osteosarcoma U2-OS cells^14^. Knocking out the RA receptor induces interdigital webbing and down-regulation of BMP7^15^. We previously reported that pretreatment with 9cRA selectively increased BMP7 mRNA expression, reduced brain infarction, and attenuated Terminal deoxynucleotidyl transferase dUTP nick end labeling (TUNEL) labeling in stroke brains^16^. These protective effects of 9cRA were antagonized by the BMP antagonist noggin^16^. A similar protective effect of 9cRA was found in an animal model of Parkinson’s disease. Early treatment with 9cRA protects against the dopaminergic neurotoxin 6-hydroxydopamine or methamphetamine-mediated neurodegeneration in nigrostriatal dopaminergic neurons^17, 18^. 9cRA reduced both methamphetamine -mediated translocation of Nurr-77 from the nucleus to cytosol, a pre-apoptotic reaction, and dopaminergic neurodegeneration. The protective activity of 9cRA against methamphetamine was antagonized by noggin^17^. Taken together, these data suggest that 9cRA can induce trophic responses, at least, through activation of BMP7. Since BMP7 has a neuroreparative effect in ischemic brain^19^, it is possible that 9cRA can also induce neurorepair through BMP7 after stroke.

In the present study, we examined the neuroreparative role of 9cRA in experimental stroke rats. Intranasal administration of 9cRA, given from 3 days after ischemic brain insult, reduced neurological deficits and increased BrdU, nestin (a neuroectodermal stem cell marker) and NeuN (a neuronal marker) immunoreactivity in the peri-lesioned cortex. Our data show that 9cRA has neuroreparative effect in stroke animals and the functional recovery may relate to the *de novo* neurogenesis in the lesioned brain.

## Results

### Separation of animals into two groups by the magnetic resonance imaging (MRI) before drug treatment

Stroke rats were separated into 2 groups with similar lesion sizes, measured by T2 -weighted imaging (T2Wi) on day 2 after dMCAo. The groups were subsequently treated intranasally with 9cRA or vehicle from day 3. The size of the lesion was limited to the right cerebral cortex (Supplement Fig. 1). Lesion volume was not different before 9cRA or vehicle treatment (p = 0.941, t-test). A subgroup of animals (n = 12) was used to examine locomotor activity on day 3. No difference was found between these two groups before drug treatment.

### Increased 9cRA level in the stroke brain after intranasal delivery of 9cRA

**Figure 1:**
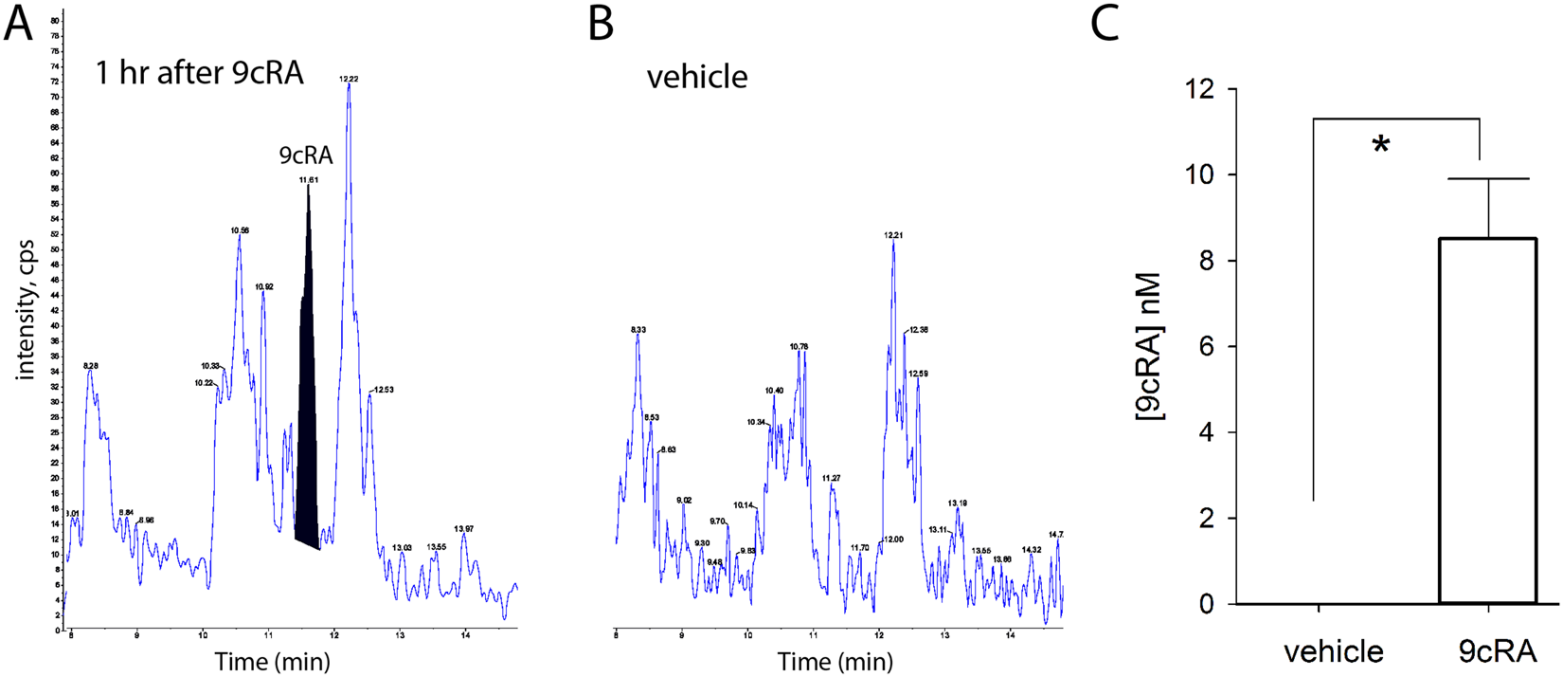
Intranasal delivery of 9cRA Increased 9cRA levels in stroke brain. Stroke rats were given either 9cRA (20 µg per animal per day, n = 3) or vehicle (n = 3) for 3 days. Brain tissues were collected at 1 hour after the last dose on day 5 for 9cRA levels measurement. Representative chromatographs of brain samples obtained from animals receiving (**A**) 9cRA or (**B**) vehicle. The retention times of the 9cRA and the internal standards were 11.5 and 7.5 min, respectively. (**C**) Intranasal delivery of 9cRA significantly increased 9cRA level in stroke brain (^*^p = 0.004).

Stroke rats received vehicle (n = 3) or 9cRA (n = 3) from day 3 to day 5 after dMCAo. Brain tissue was collected at one hour after the last dose on day 5 for LC-MS/MS analysis. Intranasal administration of 9cRA significantly increased brain 9cRA level to 8 nM (Fig. 1A and C, p = 0.004, t-test). No 9cRA was detected in the stroke brains in animals receiving vehicle treatment (Fig. 1B).

### Delayed post-stroke treatment with 9cRA improved motor behavior without affecting body weight in stroke rats

**Figure 2:**
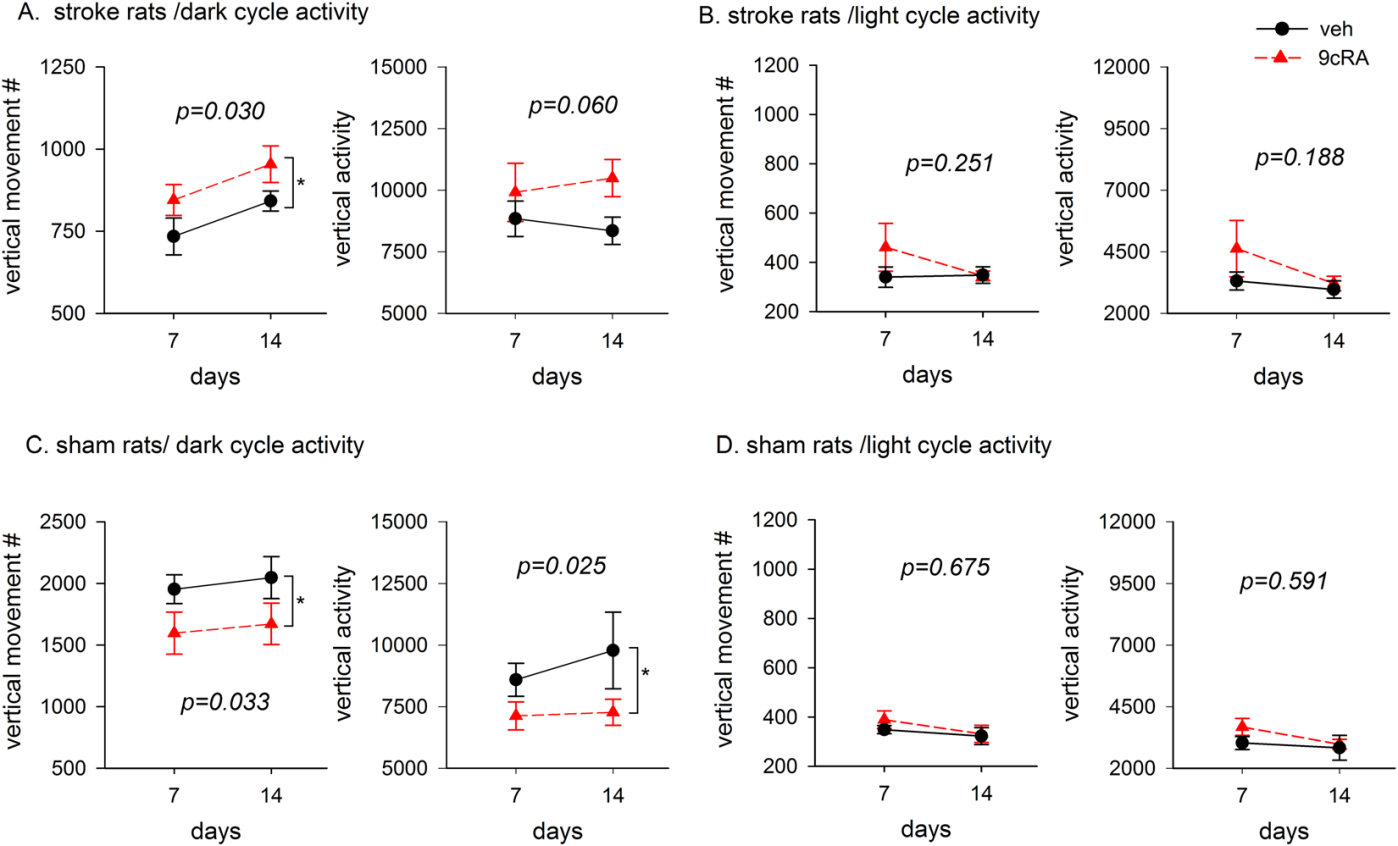
Posttreatment with 9cRA improved vertical movements during the dark cycle in stroke rats. (**A** and **B**) Stroke rats (n = 13) received intranasal treatment with 9cRA or vehicle from days 3 to 13. Animals were kept in a 12-hour light (sleep) and 12-hour dark (awake) cycle during behavioral testing on days 7 and 14. (**A**) In the dark cycle, 9cRA treatment significantly increased vertical movement numbers (p = 0.030) while marginally improving vertical activity (p = 0.060, 2-way ANOVA). (**B**) In the light cycle, 9cRA did not significantly alter vertical movement number and activity. (**C** and **D**) Another 13 rats received sham surgery on day 0 and then were treated with 9cRA or vehicle. (**C**) In the dark cycle, 9cRA significantly reduced vertical movement number (p = 0.033) and activity (p = 0.025). (**D**) These behavioral parameters were not altered by 9cRA in the light cycle. (*Significant difference).

**Figure 3:**
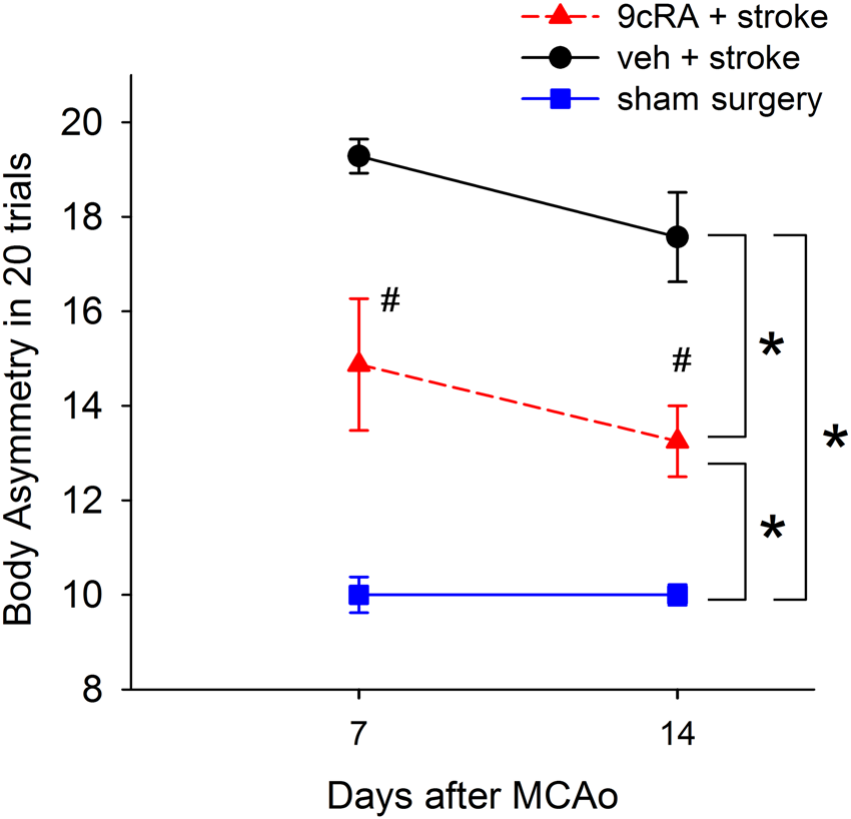
Poststroke treatment with 9cRA reduced body asymmetry in stroke rats. Stroke rats received intranasal treatment with 9cRA (n = 8) or vehicle (n = 7). An elevated body asymmetry test was used to evaluate neurological deficits on days 7 and 14. 9cRA significantly reduced body asymmetry in 20 trials (*p < 0.001, 2-way ANOVA). Posthoc Newman Keuls analysis indicates that stroke animals receiving 9cRA induced less body asymmetry than the vehicle controls on days 7 and 14 (#p < 0.001). The average body asymmetry in the sham controls (n = 7) was 10 turns/20 trials.

Two behavioral tests were used to evaluate the functional recovery after 9cRA therapy. (A) Thirteen stroke animals received intranasal vehicle or 9cRA (n = 6) or vehicle (n = 7) from day 3 to day 13. Animals were individually placed in the locomotor activity chambers for 24 hours (light cycle: 7 am to 7 pm; dark cycle: 7 pm to 7 am) on days 7 and 14. Using a two-way ANOVA, we found that the stroke animals receiving 9cRA showed enhanced recovery in motor function in the dark cycle as demonstrated by increases in vertical movement number (Fig. 2A, 9cRA vs veh, F_1,22_ = 5.366, p = 0.030; days: F_1,22_ = 5.055, p = 0.035; Interaction: F_1,22_ = 0.000, p = 0.989) and a marginal improvement in vertical activity (Fig. 2A, 9cRA vs veh, F_1,22_ = 3.917, p = 0.060; days: F_1,22_ = 0.003, p = 0.958; Interaction: F_1,22_ = 0.432, p = 0.518). In contrast, 9cRA did not alter the light-cycle vertical movement number (Fig. 2B, 9cRA vs veh, F_1,22_ = 1.388, p = 0.251; days: F_1,22_ = 1.134, p = 0.298; Interaction: F_1,22_ = 1.203, p = 0.285) and vertical activity (Fig. 2B, 9cRA vs v eh, F_1,22_ = 1.848, p = 0.188; days: F_1,22_ = 2.227, p = 0.150; Interaction: F_1,22_ = 0.711, p = 0.408). Another 13 rats receiving sham surgery were treated with 9cRA (n = 7) or vehicle (n = 6). 9cRA treatment significantly reduced vertical movement number (9cRA vs. veh, F_1,22_ = 5.152, p = 0.033; days: F_1,22_ = 0.274, p = 0.606; Interaction: F_1,22_ = 0.003, p = 0.953) and vertical activity (9cRA vs veh, F_1,21_ = 5.839, p = 0.025; days: F_1,22_ = 0.659, p = 0.426; Interaction: F_1,22_ = 0.404, p = 0.532) in the dark cycle (Fig. 2C). These vertical locomotor behavioral parameters was not affected by 9cRA in the light cycle (Fig. 2D, vertical movement number: 9cRA vs veh, F_1,22_ = 0.180, p = 0.676; days: F_1,22_ = 1.199, p = 0.285; Interaction: F_1,22_ = 0.419, p = 0.524; vertical activity: 9cRA vs veh, F_1,22_ = 0.297, p = 0.591; days: F_1,22_ = 0.096, p = 0.759; Interaction: F_1,22_ = 2.006, p = 0.171).

(B) An elevated body asymmetry test was used to evaluate neurological deficits on days 7 and 14 in 15 stroke rats. The frequency of initial turning of the head or upper body contralateral to the ischemic side was counted in 20 consecutive trials. The averaged body asymmetry in stroke animals receiving vehicle (n = 7) was 19.3 ± 0.4 contralateral turns/20 trials on day 7 an 17.6 ± 0.9 turns/20 trials on day 14 (Fig. 3). Stroke animals receiving 9cRA (n = 8) had a significant reduction in body asymmetry (Fig. 3, drug treatment, F_2,38_ = 49.358, p < 0.001; days: F_1,38_ = 2.693, p = 0.109; Interaction: F_2,38_ = 0.659, p = 0.523, two-way ANOVA). Posthoc Newman-Keuls test indicated that that stroke animals receiving 9cRA induced less body asymmetry than the vehicle controls on day 7 (p < 0.001) and day 14 (p < 0.001, Fig. 3). Another 7 rats received sham surgery on day 0 and were treated with 9cRA (n = 3) or vehicle (n = 4) from days 3 to 13. Similar to previous reports^20^, the average body asymmetry in the sham controls was 10 contralateral turns/20 trials (i.e., the animals turn in each direction with equal frequency). Since no difference was found between the sham animals receiving 9cRA or vehicle (p > 0.05), the data of all sham group animals were pooled as seen in Fig 3. Body weight was measured after 9cRA treatment. 9cRA did not significantly alter body weight in the stroke animals (veh: 262.4 ± 5.1, n = 21, vs. 9cRA: 275.7 ± 5.8 g, n = 21, p > 0.05, t test).

### Enhanced BrdU immunoreactivity in stroke rats

**Figure 4:**
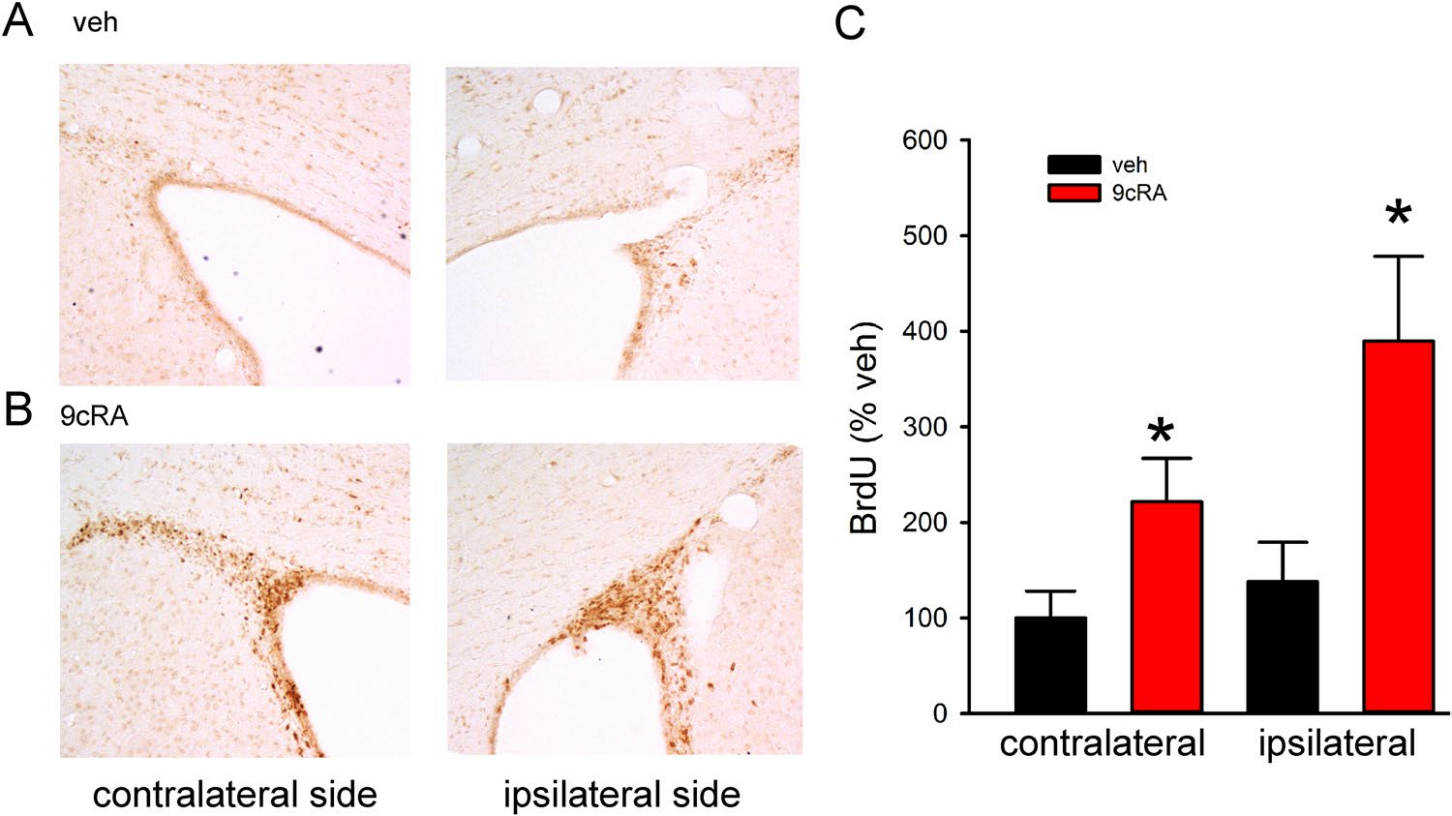
Post-stroke 9cRA treatment enhanced BrdU incorporation in SVZ. Stroke rats received intranasal treatment with 9cRA (n = 7) or vehicle (n = 6). BrdU was given on day 8. Animals were sacrificed for immunocytochemical studies on day 9. (**A**) BrdU immunoreactivity in the SVZ of a stroke animal receiving vehicle treatment. (**B**) Intranasal administration of 9cRA enhanced BrdU incorporation in the SVZ ipsilateral and contralateral to the lesion. (**C**) Post-treatment with 9cRA significantly enhanced BrdU immunoreactivity in SVZ in stroke rats (*p < 0.05, 9cRA vs veh, two-way ANOVA, n = 13).

**Figure 5:**
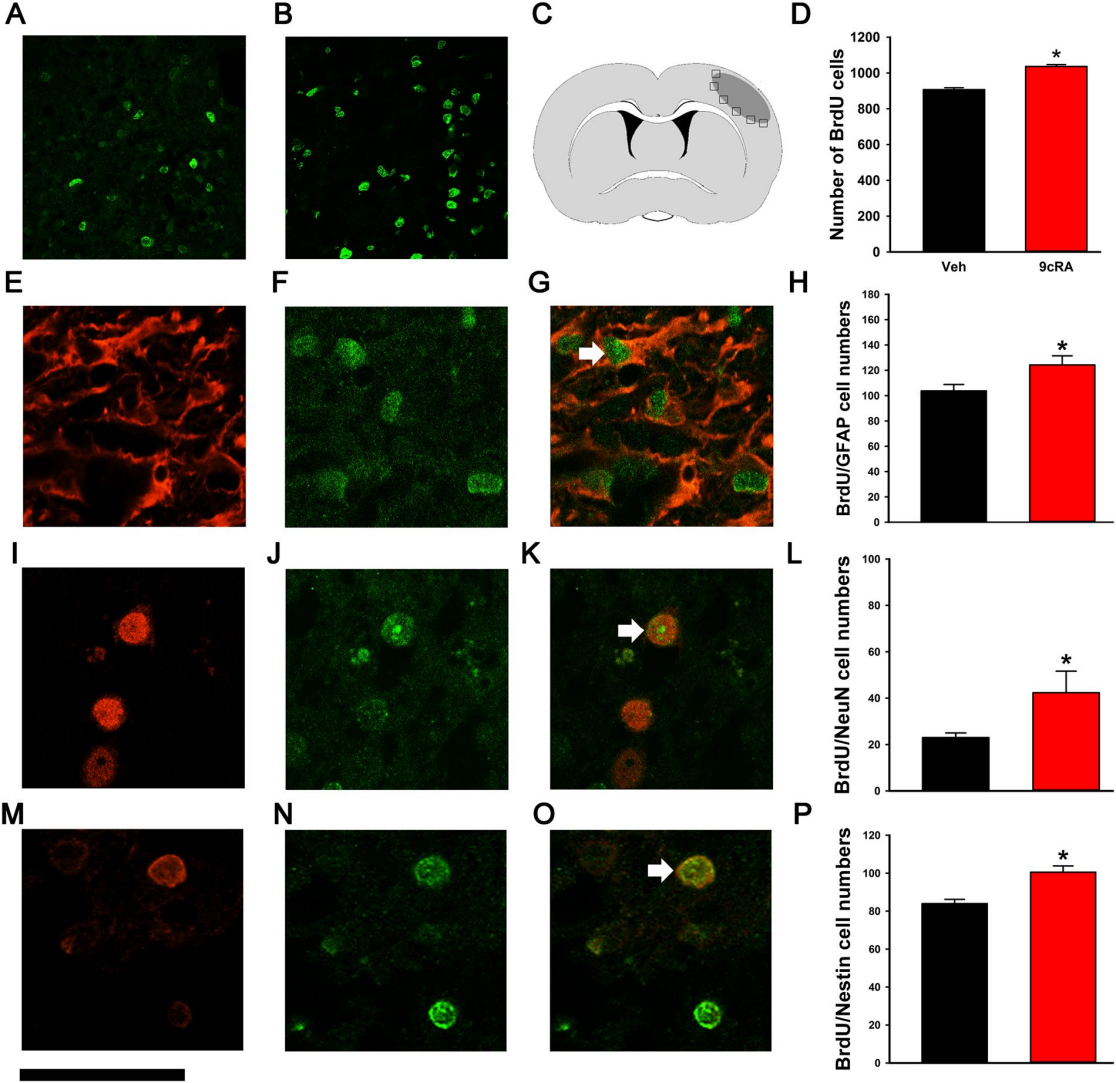
Post-stroke treatment with 9cRA increased BrdU immunoreactivity and cell differentiation in the lesioned cortex. Stroke animals (n = 13) received 9cRA (n = 6) or saline (n = 7) from days 3 to 14. BrdU was given (50 mg/kg, bid) from days 3 to 14. Animals were sacrificed on day 15. Representative BrdU immunoreactivity in the peri-lesioned area of animals receiving (**A**) vehicle and (**B**) 9cRA. The density of BrdU-labeled cells in the peri-lesioned zone (boxes surrounding the lesioned or shaded area) was measured and averaged from three adjacent sections at the level of the anterior commissure (**C**). (**D**) Intranasal administration of 9cRA significantly increased the number of BrdU-positive cells per visual field (410 × 410 µm) in the lesioned cortex (p < 0.001, t-test). Confocal microscopy was used to examine the colocalization of the immunoreactivity of BrdU (**F**,**J**,**N**) with GFAP (**E**), NeuN (**I**), or nestin (**M**) in the perilesioned cortex. The density of cells co-labelled with BrdU and these markers (arrows, **G**,**K**,**O**) were quantitatively analyzed. 9cRA treatment significantly increased the density of BrdU cells co-expressing (**H**) GFAP (p = 0.035), (**L**) NeuN (p = 0.001), and (**P**) nestin (p = 0.014). Calibration: A and B: 100 µm; E-G, I-K an M–O: 42 µm.

BrdU immunoreactivity was used to evaluate the NPC proliferation and differentiation in stroke brain. (A) In the proliferation experiment, stroke rats (n = 13) were treated with 9cRA or vehicle from days 3 to 8 after dMCAo; BrdU (4 doses) was given on day 8. Animals were sacrificed for immunocytochemical studies on day 9. Since BrdU-positive cells were tightly packed in the SVZ, BrdU optical density was obtained using NIS Elements AR 3.2 Software (Nikon) as we previously described^2, 3^. As seen in Fig. 4A and B, 9cRA treatment enhanced BrdU incorporation in the ipsilateral and contralateral SVZ. The density of BrdU-labeled immunoreactivity in the SVZ was averaged from three sections (−0.3 mm, 0.3 mm, and 1.0 mm from bregma) and normalized to the mean BrdU immunoreactivity in the contralateral SVZ of vehicle animals. 9cRA significantly enhanced BrdU immunoreactivity in SVZ in stroke rats (9cRA vs veh., F_1,22_ = 12.296, p = 0.002; ipsilateral vs contralateral v: F_1,22_ = 3.735, p = 0.066; Interaction: F_1,22_ = 1.492, p = 0.235, two-way ANOVA, Fig. 4C). The enhanced BrdU labeling in SVZ in 9cRA-treated animals suggests an increase in cell proliferation in the SVZ after ischemic insults. (B) In the cell differentiation experiment, another set of stroke animals (n = 13) were treated with 9cRA (n = 6) or vehicle (n = 7) from days 3 to 14. All animals received daily BrdU injection (50 mg/kg, bid) from days 3 to 14 and were sacrificed on day 15. The actual number of BrdU-positive cells (Fig. 5A and B) in the peri-lesioned area of cerebral cortex was counted and averaged from three adjacent sections at the level of the anterior commissure as previously described^2^ (Fig. 5C). Intranasal administration of 9cRA significantly increased the density of BrdU-positive cells in the lesioned cortex on day 15 (Fig. 5A,B and D, p < 0.001, t-test). The differentiation of NPCs in the lesioned cortex was examined using double-label immunostaining. BrdU-positive cells (Fig. 5F,J and N) in the lesioned side cerebral cortex expressed the glial marker glial fibrillary acidic protein (GFAP, Fig. 5E), neuron-specific nuclear protein (NeuN, Fig. 5I), and neuronal stem cell markers nestin (Fig. 5M). 9cRA treatment significantly increased the density of BrdU (+) cells coexpressing nestin GFAP (p = 0.035, Mann-Whitney rank sum test, Fig. 5G and H), Nestin (p = 0.001, Mann-Whitney rank sum test, Fig. 5K and L), and NeuN (p = 0.014, Mann-Whitney rank sum test, Fig. 5O and P).

### 9cRA increased NPC proliferation in SVZ culture

**Figure 6:**
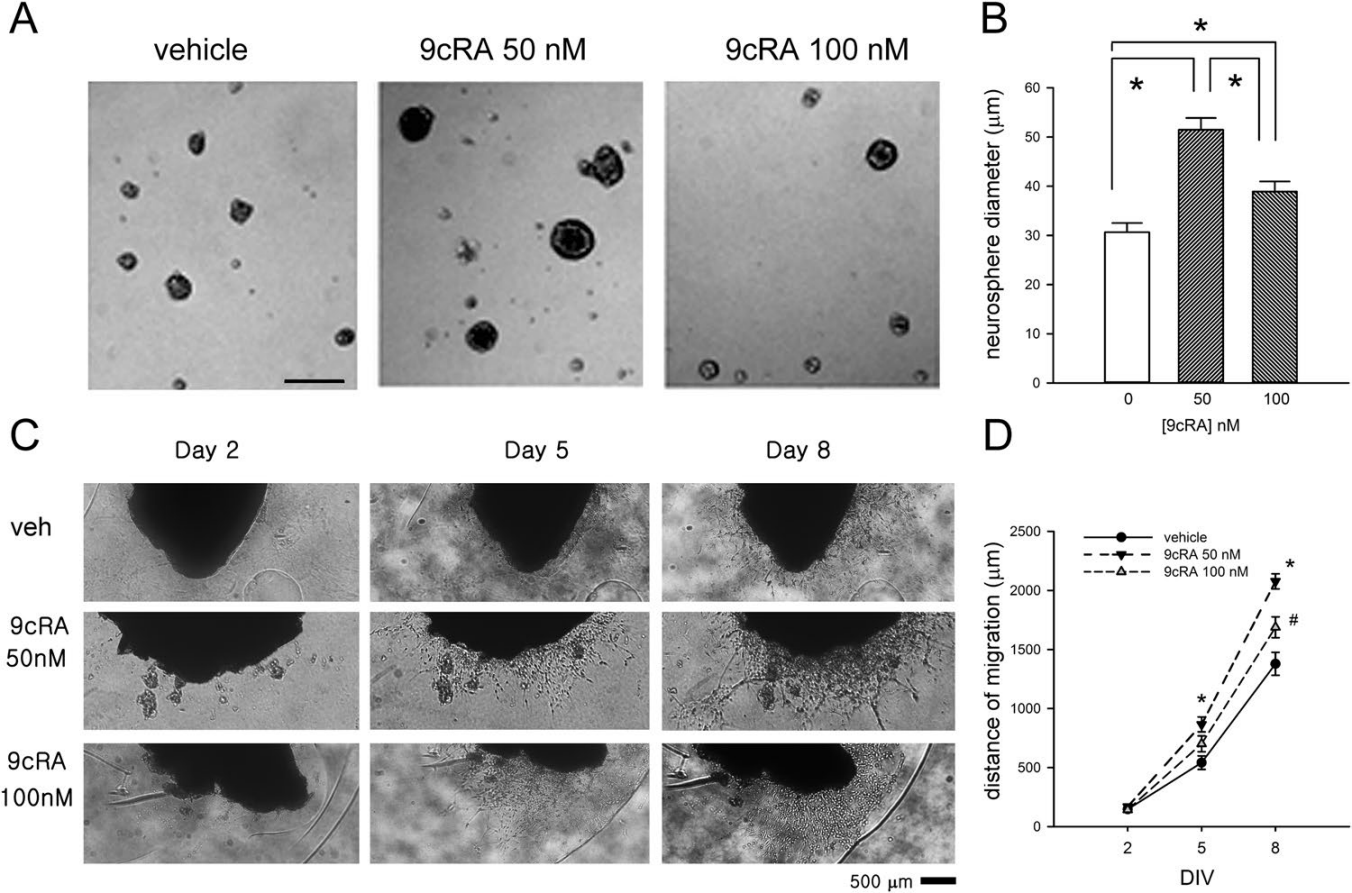
9cRA increased proliferation and migration of SVZ cells in culture. (**A**) 9cRA increased the diameter of SVZ neurosphere. (**B**) 9cRA, at 50 nM, significantly enhanced diameter of neurosphere (^*^p < 0.05, one way ANOVA on Rank + posthoc Dunn’s test). Calibration = 100 µm. (**C**) The migration of SVZ cells was examined in the matrigel culture. 9cRA increased the cell migration on DIVs 5 and 8. (**D**) Treatment with 9cRA significantly enhanced the distance of cell migration (^*,#^p < 0.002, two-way ANOVA + Newman-Keuls test).

SVZ cells were collected from adult rat brains and cultured in growth media containing basic fibroblast growth factor (bFGF) and epidermal growth factor (EGF) to form neurosphere (spherical floating clusters of neural stem cells). As seen in Fig 6A, 9cRA increased the size of SVZ –derived neurospheres. A significant difference in neurosphere diameters was observed in cells receiving 9cRA on DIV (days *in vitro*) 8 (Fig. 6B, p < 0.001, H = 39.793, 1-way ANOVA on Ranks). Low-dose 9cRA (50 nM) was more efficient to increase the size of neurospheres, as compared to a higher dose of 100 nM (Fig. 6B, p < 0.05, Q = 6.303, posthoc Dunn’s test).

### 9cRA enhanced cell migration from subventricular zone (SVZ) explants

The 9cRA -induced cell migration from SVZ explants was examined in the Matrigel cultures (Fig. 6C and D). The cultured SVZ explants were treated with 9cRA or vehicle. The distance of SVZ cell migration from the explants was examined under a microscope from DIV 1 to 8. There was minimal cell migration before DIV 2 (Fig. 6C,D). Treatment with 9cRA significantly enhanced the distance of cell migration on DIVs 5 and 8, comparing to the vehicle controls (Fig. 6D, 9cRA: F_2,135_ = 24.647, p < 0.001; days: F_2,135_ = 508.315, p < 0.001; interaction: F_4,135_ = 7.772, p < 0.001, two-way ANOVA). Newman-Keuls posthoc test indicates significant difference between 50 nM 9cRA and veh (q = 9.909, p < 0.001), 50 nM 9cRA and 100 nM 9cRA (q = 5.498, p < 0.001), and 100 nM 9cRA and veh (q = 4.411, p = 0.002).

### 9cRA enhanced the expression of BMP7/noggin in SVZ of stroke rats

**Figure 7:**
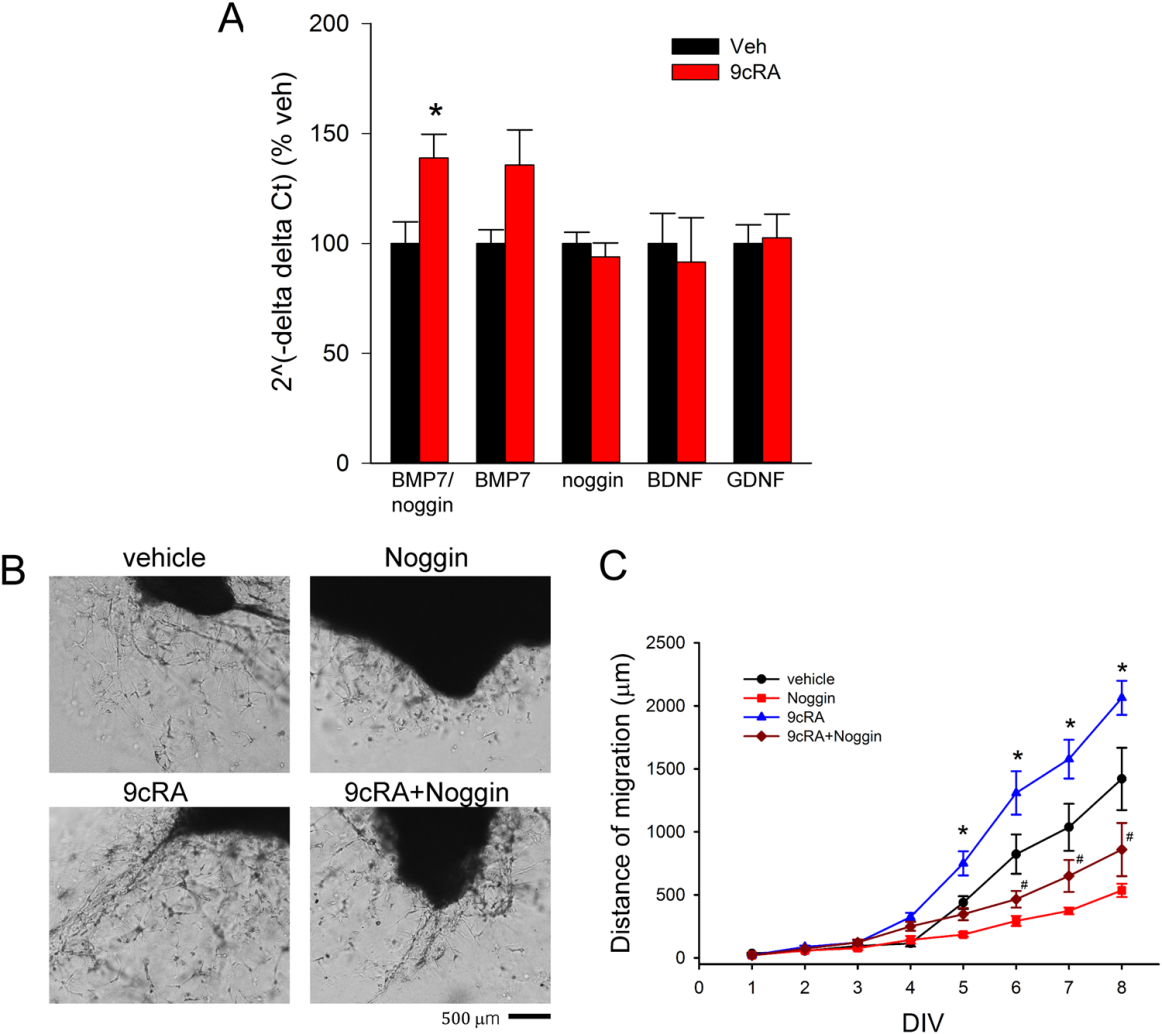
9cRA -enhanced BMP7 expression and migration of SVZ cells (**A**) SVZ tissues were collected from stroke rats receiving intranasal 9cRA (n = 9) or vehicle (n = 9). The expression of BMP7, noggin, GDNF, and BDNF was examined by qRT-PCR and normalized to reference genes (GAPDH and actin). (**A**) 9cRA significantly enhanced the expression of BMP7/noggin (p = 0.0167, t-test). The expression of GDNF or BDNF in the SVZ was not affected by intranasal 9cRA. (p > 0.5, A &B). (**B**) SVZ explants were treated with 9cRA or vehicle from DIV2 for one week. 9cRA, at 50 nM, significantly enhanced cell migration from the SVZ explants. Co-treatment with noggin reduced cell migration from SVZ explants. (**C**) Noggin significantly antagonized 9cRA -enhanced cell migration from SVZ explants (^*,#^p < 0.01, two-way ANOVA + Newman-Keuls test).

A total of 18 rats received intranasal 9cRA (n = 9) or vehicle (n = 9) from days 3 to 6 after dMCAo. SVZ tissues were collected on day 7 to examine the expression of BMP7, noggin, GDNF, and BDNF by qRT-PCR. Relative gene expression was obtained by normalization with two reference genes GAPDH and actin. 9cRA marginally increased BMP7 expression (p = 0.052, Mann-Whitney Rank Sum Test, Fig. 7A). 9cRA did not alter noggin, GDNF, and BDNF, expression (p > 0.5, t-test, Fig. 7A). Since BMP7 and its antagonist noggin play an integral role during development or injury^21, 22^, we examined the BMP7/noggin ratio after 9cRA or vehicle treatment. The ratio of BMP7 to noggin in the SVZ was significantly increased by 9cRA (Fig. 7A, p = 0.0167, t-test).

### 9cRA enhanced migration of SVZ cells through BMP7

We next examined if BMP7 was involved in 9cRA -mediated migration of SVZ cells. Noggin was co-administrated to the matrigel culture at 100 ng/mL to inhibit BMP as previously described^23^. The addition of noggin significantly reduced 9cRA -enhanced cell migration from SVZ explants (Fig. 7B and C, drug:, F_3,254_ = 62.569, p < 0.001; days:, F_7,254_ = 83.968, p < 0.001; interaction: F_21,254_ = 9.528, two-way ANOVA). Posthoc Newman-Keuls test indicates that noggin significantly reduced 9cRA –mediated cell migration (9cRA vs. 9cRA + noggin, q = 14.859, p < 0.001). These data suggest that BMP is involved in 9cRA-induced SVZ cell migration.

## Discussion

Focal cerebral ischemia causes neurological and motor deficits in experimental animals. The impairment in body asymmetry and vertical locomotor movement can last for weeks after stroke in rats^24^. We demonstrated that animals receiving vehicle had a significant increase in body asymmetry at one and two weeks after a 90-min dMCAo. Post-stroke treatment with 9cRA from days 3 to 13 significantly reduced body asymmetry. Locomotor activity was monitored during 12 hour- light (sleep) and 12 hour –dark (awake) cycle on days 7 and 14. 9cRA significantly improved vertical movement in the dark cycle when animals were most active. These data suggest that 9cRA reduced neurological symptoms and improved locomotor function in stroke animals. 9cRA did not alter body weight and locomotor activity in the light cycle when animals were asleep, which indirectly suggest that appetite or sleep was less affected by 9cRA in stroke animals. Along with the behavioral improvement, 9cRA enhanced the proliferation, migration, and differentiation of SVZ BrdU(+) cells in stroke brain. 9cRA also increased the proliferation and migration of SVZ cells in culture. Our data suggest that post-stroke treatment with 9cRA induces neurorepair in the ischemic brain.

After the onset of ischemic brain injury, a series of time-dependent pathophysiological responses are activated^25^. Some of these reactions occur shortly after and last only for hours to days after stroke. For example, cerebral infarction^26, 27^ and TUNEL^2^ in the ischemic cortex peak on days 1 or 2 after dMCAo. In current study, brain infarction was examined by MRI on day 2 after dMCAo. Similar sized infarction and behavioral deficits were found in the 9cRA versus vehicle control groups before drug treatment. The improvement of motor activity after the delayed 9cRA treatment may not be attributed to the reduction of infarction in the ischemic cortex. However, the death-signals were not limited to the lesioned site. TUNEL and selective death genes can be activated in the SVZ at 10 days after dMCAo^2^. The anti-apoptotic drug PFTα suppressed cell death and prolonged the survival of SVZ cells^2^. It is possible that delayed 9cRA treatment also improved survival of SVZ cells through anti-apoptotic mechanisms in the SVZ^13, 16, 17^. These possibilities will require further investigation.

The survival, proliferation or migration of neuroprogenitor cells from the SVZ can be regulated by several trophic factors. Loss of neurotrophic factor activity is a major contributor to post-ischemic degradation of the neural microenvironment^28^. For example, increasing brain -derived neurotrophic factor (BDNF) expression in the SVZ through AAV infection^29^, intracerebral administration of BMP7^19^, or glial cell line -derived neurotrophic factor (GDNF)^30^ enhanced proliferation and migration of NPCs from the SVZ in stroke animals. To characterize the downstream mechanism(s) of 9cRA, we examined that these trophic factors in the SVZ after drug treatment. 9cRA marginally enhanced the expression of BMP7, but not GDNF or BDNF, in the SVZ of stroke rats. Noggin is an endogenous cystine-knot protein which binds BMP7, blocks the binding epitopes to the BMP receptors^31^, and neutralizes BMP7 actions. Both BMP7 and noggin are present in the subependymal layer in brain^32^. Previous studies have shown that the expression of BMP7 and its antagonist noggin can be simultaneously regulated by insults^33^ and play an integral role during development or injury^21, 22^. BMP7 also modulates noggin expression during development^34^. The physiological responses associated with BMP7 upregulation can be altered by the co-expression of noggin. To minimize confounding interactions, we examined the BMP7/noggin ratio after 9cRA treatment. The ratio of BMP7 to noggin in the SVZ was increased by 9cRA. These data support that 9cRA differentially upregulated BMP7 expression in the SVZ. Since the change in protein did not always correlate with the mRNA expression, further protein analysis is required when specific antibodies are available.

The antagonistic action of noggin against BMP7 has been documented in stroke brain. Ischemic preconditioning, fetal kidney transplantation^20^, or pretreatment with 9cRA upregulated the expression of BMP7 and attenuated brain infarction^35^; these protective effects were antagonized by noggin^35, 36^. In our current study, we found 9cRA increased the expression of BMP7/noggin and noggin antagonized 9cRA - mediated cell migration from the SVZ explants, indicating 9cRA enhanced cell migration through BMP –mediated mechanism. Noggin also reduced the basal cell migration in control SVZ explants, suggesting that endogenous BMP also participates in the basal cell migration.

9cRA -mediated neuroregeneration through BMP7 in stroke brain is further supported by the similar neuroreparative action of BMP7. Post-treatment with BMP7 enhanced recovery of sensorimotor function in the impaired limbs^37, 38^, decreased body asymmetry and increased in locomotor activity from days 7 to 14 after stroke^39^. BMP7 promoted DNA synthesis as visualized by BrdU incorporation in cultured mesencephalic neurons^40^ and in the SVZ of stroke rats^19^. These data support that 9cRA or BMP7 improve functional recovery through the proliferation of new neuronal precursors in the stroke brain.

Besides its interaction with BMP7, 9cRA may induce trophic responses in stroke brain through other pathways. For example, 9cRA is a potent antioxidant and can suppress the inflammatory responses in cultured microglia and astrocytes^41^. RA inhibited H_2_O_2_-induced apoptosis via suppression of c-fos/c-jun expression and c-Jun N-terminal protein kinase (JNK) activation in mesangial cells^42^. 9cRA inhibited the export of Nurr77 from the nucleus to cytosol, a response that activates apoptosis after injury^17^ and suppressed apoptosis in stroke rats^16^. 9cRA can also upregulate other neuroprotective trophic factors, such as midkine^13^. It is possible that multiple mechanisms are involved in the neuroreparative action of 9cRA and further investigation is warranted.

BrdU, a thymidine analog that can be incorporated into DNA during the S-phase of the cell cycle, was used to examine NPC proliferation and differentiation after stroke. In the current proliferation study, 4 injections of BrdU were given to the stroke rats on day 8. Treatment with 9cRA significantly enhanced BrdU incorporation in the SVZ on day 9, suggesting that 9cRA increased cell proliferation in the SVZ after ischemic insults. We previously demonstrated that the BrdU-positive cells migrated from the SVZ to the lesioned area between 10 and 21 days after dMCAo^2^. A significant correlation was found between the functional outcomes and number of surviving BrdU-positive cells in the lesioned cortex^2^. To examine the differentiation of NPCs in the lesioned cortex, stroke rats received BrdU daily from days 3 to 14 and were sacrificed on day 15. 9cRA significantly increased the density of BrdU-positive cells and the co-expression of nestin, NeuN, and GFAP with BrdU in the lesioned cortex. Taken together, these data suggest that 9cRA promotes the migration/differentiation of these cells in the lesioned site.

9cRA was administered intranasally in this study. This approach is a minimally invasive way to repeatedly deliver drugs to stroke animals. Although blood-brain-barrier is transiently compromised after MCAo, it can still limit the exogenous compounds entering brain parenchyma at weeks after stroke. Small molecules, given intranasally, can pass through the blood-brain barrier, avoid first pass metabolism, and reduce non-selective effects in the periphery. We demonstrated that 9cRA, given intranasally, increased 9cRA levels in the stroke brain. 9cRA was not found in animals receiving vehicle. 9cRA was not detectable in mouse embryos^43–45^ or tissue extracts from adult rats^46^. The increase in 9cRA in stroke brain in the present study was thus mainly derived from the intranasal 9cRA. Previous studies have indicated that 9cRA binds and activates RXR with high affinity (Kd = 9–12 nM^7^ or 14.1–15.7 nM^47^). 9cRA enhances RXR homodimer formation at 10^–9^ to 10^–8^ M, leading to the activation of several response elements^48^. In our study, intranasal administration of 9cRA increased brain 9cRA levels to 8.3 nM at one hour after injection. A higher concentration of 9cRA in brain may occur within one hour after drug delivery. It is likely that the dose of 9cRA used in this study was able to activate RXR receptors in brain.

Increasing evidence has supported that ischemic stroke induces neuroprogenitor cell proliferation in the SVZ of experimental animals^2, 49, 50^. This effect has also been demonstrated in human patients. Ischemic injury increased the NPC marker polysialylated neural adhesion cell molecule (PSA-NCAM) in the postmortem adult human SVZ tissue^51^. Another study examined the SVZ in elderly patients who died 5–21 days after onset of ischemic stroke. There was a 6-fold increase in PSA-NCAM and a 2-fold increase in Ki-67 (a proliferation marker) cell numbers in the ipsilateral SVZ, suggesting ischemic insults can increase proliferation and neuroblast formation in the SVZ in stroke patients^52^. Similar findings were also reported in patients following subarachnoid hemorrhage (SAH)^53^. CNS progenitor cell markers, such as nestin, Musashi −1 and −2, were upregulated in the cerebral tissues of patients with SAH. These data suggest that activation of neuroprogenitor cells proliferation occurs in the adult human brain after ischemic or hemorrhage insults. In this study, we demonstrated that intranasal administration of 9cRA increased proliferation, migration, and differentiation of SVZ cells in stroke rats. The use of 9cRA is potentially useful to enhance the survival of SVZ cells and improve the behavioral outcome in stroke patients.

We and others have previously demonstrated that pretreatment with 9cRA reduced cerebral infarction in stroke rats^13^. In current study, we further identified that post-treatment with 9cRA, given after the peak of cerebral infarction (i.e. from the 3^rd^ day after dMCAo), attenuated neurological deficits and improved locomotor behavior. The reparative action of 9cRA is associated with proliferation, migration, and differentiation of NPCs. As neuroinflammation can continue to occur in the peri-infarct brain regions for days after ischemia^54^, other mechanisms, such as the interaction with the delayed neuroinflammation or degeneration, may also be involved in the behavioral recovery after 9cRA therapy and remain to be identified. In summary, we demonstrated that delayed intranasal treatment with 9cRA modifies endogenous neural repair in stroke brain and improves functional recovery. Our results may provide a new treatment strategy for stroke patients, enabling a non-invasive and longer treatment window of days after stroke occurrence.

## Methods

### Animal and surgery

Adult male Sprague-Dawley rats (3 months old) were used for this study. All study protocols were approved by the Animal Research Committee at the National Health Research Institutes (approved # 102068 and 105080) and the National Institute on Drug Abuse (approved # CNRB-77). All animals were treated in accordance with the Guide for the Care and Use of Laboratory Animals published by the US National Institutes of Health). Animals were anesthetized with chloral hydrate (400 mg/kg, i.p.). A craniotomy of about 2–4 mm^2^ was made in the right squamosal bone. The right distal brain of MCA was ligated (dMCAo) with a 10-O suture using methods previously described^27, 55^. After 90 min of ischemia, the suture on the MCA and arterial clips on common carotids were removed to allow reperfusion. Core body temperature was monitored and maintained at 37 °C. After recovery from the anesthesia, body temperature was still maintained at 37 °C using a temperature-controlled incubator. Control animals received sham surgery including craniotomy without dMCAo. At the end of experiment, all animals were euthanized by CO_2_ or decapitation.

### MRI

All rats underwent MRI scanning 2 days after dMCAo under isoflurane anesthesia. MRI experiments were performed on a Bruker BioSpin 9.4 T animal MRI scanner (Bruker Medizintechnik, Karlsruhe, Germany) as previously described^56^. Ischemic injury size was determined from the hyper-intensity region in the T2Wi.

### Intranasal administration of 9cRA or vehicle

9cRA or vehicle (10% DMSO in saline) was given intranasally from day 3. Rats were anesthetized with isoflurane and were placed in a supine position. 9cRA or vehicle was delivered into nostrils of each rat at a dose of 20 μl daily as previously described^3^.

### Measurement of 9cRA level in brain

(i) Tissue processing and extraction: The analysis of 9cRA in brain was performed by Absorption Systems LP (Exton, PA). Stroke rats were treated with 9cRA or vehicle intranasally from days 3 to 5. Brain tissues were collected at 1 hour after the last drug delivery on day 5. Tissue samples were stored frozen at −80 °C until analysis. The tissues were thawed, weighed, and homogenized in 3 volumes of phosphate buffered saline (PBS) using an ultrasonic tissue disrupter. The samples remained on ice at all times during the homogenization process and the process was performed under yellow light. For calculation of the grams of tissue in the homogenate, the tissue weight was assumed to have a density of water. The total volume of the homogenate was 1 volume tissue +3 volumes PBS for a total of 4 volumes. The analytes were extracted from the homogenates using a liquid/liquid extraction described in Kane *et al*.^57^. Since the tissue may contain many retinoids which interfere with the analysis of the retinoic acids, the neutral retinoids were removed from the tissues by extraction into hexane under basic conditions. A 0.5 mL aliquot of the tissue homogenate was basified with 1 mL of ethanolic KOH (0.025 M), the internal standard was added (50 uL of all-trans-acitretin at 100 ng/mL), and the neutral retinoids extracted into 2 × 5 mL of hexane. The hexane was discarded and the remaining aqueous layer was acidified using 65 µL of 4 N HCl. After mixing, the retinoic acids were extracted with 2 × 5 mL of hexane. The hexane was evaporated to dryness under a stream of nitrogen at 40 °C. The dried samples were reconstituted in 150 µL of acetonitrile. All extraction steps were performed under yellow light.
(ii) Chromatography: To separate the 9-cis-retinoic acid from its isomers, 13-cisretinoic acid and all-trans-retinoic acid, a separation method from Kane *et al*.^57^, was modified to separate the isomers. The separation is achieved using a 2.1 × 150 mm Symmetry C18 column with a gradient program using ACN:MeOH:H2O:formic acid (40:30:30:0.1) as mobile phase A (MPA) and ACN:MeOH:H2O:formic acid (55:30:15:0.1) as mobile phase B (MPB). The flow rate was set at 0.25 mL/min and the column. The retention times of the 9-cRA, all-trans-retinoic acid and the internal standard were 11.5, 12.2, and 7.5 min, respectively.

### Administration of BrdU

Two BrdU injection protocols were used: (1) BrdU (Sigma-Aldrich, St Louis, MO) was administered on day 8 (50 mg/kg × 4, i.p. at a 2-hour interval). Animals were sacrificed on day 9 to examine the proliferation of NPCs in SVZ. (2) BrdU was given daily (50 mg/kg, bid, i.p.) from days 3 to 9 post-dMCAo. Brain tissues were analyzed for the migration and differentiation of NPCs in the lesioned cortex.

### Behavioral measurements

Stroke animals received two behavioral tests: (A) Locomotor activity. Each animal was placed in a 42 × 42 × 31 cm open plexiglass box. Locomotor activity was recorded with an Accuscan activity monitor (Accuscan, Columbus, OH) for 24 hours (12-h light and 12-h dark/day) on days 7 and 14 after dMCAo or sham surgery as previously described^58^. The monitor contained 8 vertical infrared sensors situated 10 cm from the floor of the chamber. Motor activities were calculated by the number of beams broken for 24 hours after placement in the chamber. The animals were able to lift their forearms for rearing, drinking, and taking food. Vertical movement number (the total number of rearing up as detected by the vertical sensors) and vertical activity (the total number of beam interruptions that occurred in the vertical sensors) were analyzed by the Versamax program (Accuscan, Columbus, OH).

(B) Body asymmetry was analyzed using an elevated body asymmetry test^59^. Rats were examined for lateral movements/turning when their bodies were suspended 20 cm above the testing table by lifting their tails. The frequency of initial turning of the head or upper body contralateral to the ischemic side was counted in 20 consecutive trials. The maximum impairment in body asymmetry in stroke animals is 20 contralateral turns/20 trials. In non-stroke rats, the averaged body asymmetry is 10 contralateral turns/20 trials (i.e., the animals turn in each direction with equal frequency).

### Immunohistochemistry

Animals were anesthetized with chloral hydrate (400 mg/kg i.p.) and perfused transcardially with saline followed by 4% paraformaldehyde (PFA) in phosphate buffer (PB; 0.1 M; pH 7.2). The brains were dissected, post-fixed in PFA for 48–72 hours, and transferred to 20% sucrose in 0.1 M PB for at least 16 hours. Serial sections of the entire brain were cut at 35 µm thickness on a freezing cryostat (Leicmea, Model: CM 3050 S). One series of sections from every 6 sections was stained for each antibody used.

Sections were rinsed in 0.1 M PB; DNA was denatured with 50% formamide (Biofluids Division, BSI, Rockville, MD) at 65 °C for 2 hours in a water bath. Brain sections were incubated in dH2O containing 2 N HCl at 37 °C for 30 min and rinsed with boric acid (0.1 M, pH 8.5) for 10 min and then with PB at RT. After blocking with 4% bovine serum albumin (BSA) and 0.5% Triton x-100 in 0.1 M PB, brain slices were incubated with primary antibodies against BrdU (monoclonal 1:500, Sigma-Aldrich, USA), NeuN (rabbit monoclonal, 1:200, Millipore, MA), Nestin (1:200, Abcam, Cambridge, MA), or GFAP (rabbit monoclonal, 1:500, Millipore, MA) for 24 hour at 4 °C. Slices were washed three times with 0.1 M PB and incubated in Alexa Fluor 488 goat anti-mouse (1:500, Molecular Probes, Eugene, OR) or Alexa Fluor 568 goat anti-rabbit IgG antibody solution (1:500, Molecular Probes, Eugene, OR) for 90 min at RT. Control sections were incubated without primary antibody. Sections were mounted on slides and cover-slipped.

Confocal analysis was performed using a Nikon D-ECLIPSE 80i microscope (Melville, NY, USA) and EZ-C1 3.90 software. The optical density with each antibody immunoreactivities was quantified in three consecutive brain sections with a visualized anterior commissure in each animal. Six photomicrographs were taken along the perilesioned region per brain slices (see Fig. 6D); BrdU optical cell density was analyzed by NIS Elements AR 3.2 Software (Nikon) and was averaged in each brain for statistical analysis. All immunohistochemical measurements were done by blinded observers.

### SVZ neurosphere cultures

SVZ cells were collected from adult rat brains and cultured as neurospheres as described previously^3^. Cells were plated in 24-well plates in 10% DMEM/F12 medium supplemented with 2% B27 supplement, 2 mM L-glutamine, 0.5% HEPES, 1% Pen/Strep, 0.02% heparin, EGF (40 ng/mL), and bFGF (10 ng/mL). 9cRA or vehicle was added from DIV1 to DIV7 (days *in vitro*). The sizes of neurospheres were analyzed on DIV8.

### SVZ explant culture

SVZ explant cultures were prepared from adult male rats. SVZ explants were cultured within Matrigel (BD Biosciences) in Neural basal-A medium containing 2% B27 supplement (Invitrogen). The cultured SVZ explants were treated with 9cRA or vehicle on days 1, 2 and 3 and some cultured with noggin (100 ng/mL, R&D, Minneapolis, MN) on days 2–4 as previously described^23^. The distance of SVZ cell migration was examined from days 2–7 after culture.

### Quantitative Reverse Transcription –PCR (qRTPCR)

Stroke (n = 18) and control (sham surgery, n = 10) rats received intranasal 9cRA or vehicle from days 3 to day 6. SVZ tissues were collected on day 7 for qRTPCR analysis as previously described. Total RNAs were isolated using TRIZOL Reagents (Life Technologies, #15596–026) and cDNAs were synthesized from 1ug total RNA using a RevertAid H Minus First Strand cDNA Synthesis Kit (Thermo Scientific, #K1631). The TaqMan Gene Expression Assays (primer and probe set) for specifically detecting rat BMP7 (#Rn01528889_m1), noggin (#Rn01467399_s1) and beta-actin (Rn00667869_m1) were purchased from Thermo Scientific. Primers and 6-carboxyfluorescein (FAM) -labeled probes used in the quantitative RT-PCR for other trophic factors are as follows: BDNF forward primer (5′-ACTTTTGAGCACGTCATCG); reverse primer (5′-TCCTTATGGTTTTCTTCGTTGG); probe (mouse/rat universal probe library No. 42; Roche); GDNF forward primer (5′-TAAGATGAAGTTATGGGATGTCG); reverse primer (5′-CTTCGAGAAGCCTCTTACCG); probe (mouse/rat universal probe Library #112; Roche). Quantitative Real-Time PCR (qRT-PCR) was carried out using TaqMan Fast Advanced Master Mix (Life Technologies, #4444557) and Applied Biosystems 7500 Fast Real-Time PCR System.

Nine biological and 2 technical replicates were examined in the stroke animals; five biological replicates and 2 technical replicates were examined in the control group. The expression of the target genes (delta-Ct) was analyzed by comparing to two reference genes (GAPDH and beta-actin) using the Applied Biosystems 7500 Real-Time PCR Software (v 2.0.6). 9cRA -mediated changes in gene expression (2^−(delta-delta-Ct)^) was further normalized to the vehicle group.

### Statistical analysis

Values are means ± s.e.m. Unpaired 2 tailed t-tests, Mann-Whitney Rank Sum test, 1- or 2-way ANOVA were used for statistical analysis. The Kruskal-Wallis ANOVA analysis on ranks was used when the normality assumption was violated. Post hoc Newman-Keuls or Dunn’s test was used for all multiple pairwise comparisons. All statistics were performed using Sigmaplot software (Systat Software, Inc.). A statistically significant difference was defined as p < 0.05.

## Electronic supplementary material

Supplementary Information
