# Supplementary material for: 9-cis retinoic acid induces neurorepair in stroke brain
Source: Sci Rep. 2017 Jul 3;7:4512. doi: 10.1038/s41598-017-04048-2 (PMC5495771; doi:10.1038/s41598-017-04048-2)
Supplement: Supplementary file 1 — Supplementary Information [file 41598_2017_4048_MOESM1_ESM.doc]

**Supplemental Title page**

**Manuscript title: 9-cis retinoic acid induces neurorepair in stroke brain**

Authors: *Seong-Jin Yu^1^, ^#^Mikko Airavaara^2^, Kuo-Jen Wu^1^, Brandon K Harvey^3^, H.S. Liu^3^, Yihong Yang^3^, Alex Zacharek^4^, Jieli Chen^4^, Yun Wang^1^,*

^1^Center for Neuropsychiatric Research, National Health Research Institutes, Taiwan;

^2^Institute of Biotechnology, Viikki Biocenter, University of Helsinki, Helsinki, Finland

^3^ Intramural Research Program, National Institute on Drug Abuse, NIH, USA

^4^Neurology, Henry Ford Hospital, Detroit, MI, USA

**Supplemental Figure 1:**


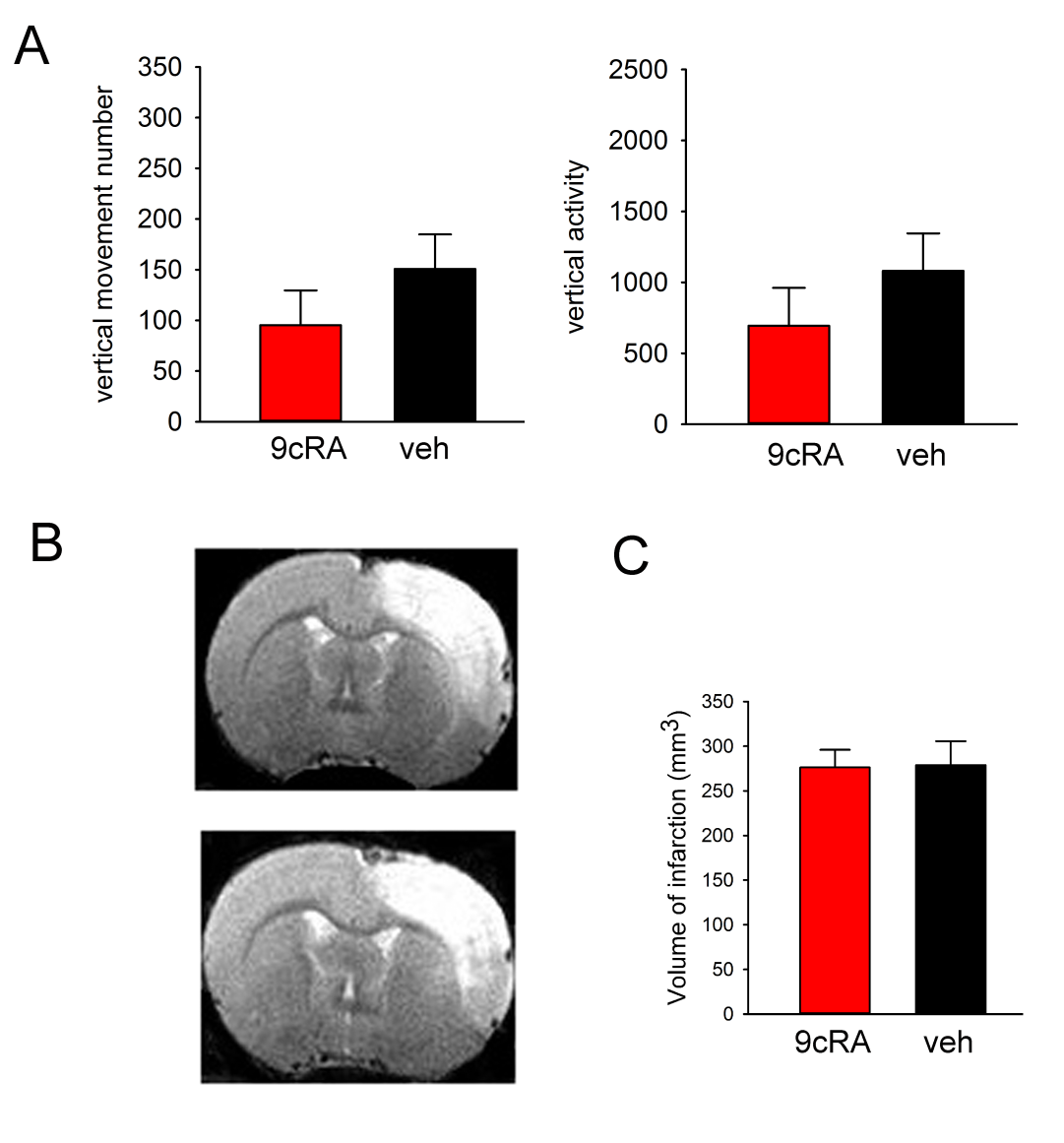


**Supplemental Figure 1**: **Separation of animals to two groups before drug treatment.** Stroke animals were separated into 2 groups to have similar lesion sizes, measured by T2Wi on day 2 before receiving 9cRA or vehicle and were used for behavioral and histological measurement after drug treatment. (A) No difference in 1-hour locomotor activity was found between these two groups before drug treatment (n=12). (B) The lesion (infarction, arrow) was limited to the right cerebral cortex. (C) Lesion volume was not different before 9cRA or vehicle treatment (p=0.941, n=41, t-test).


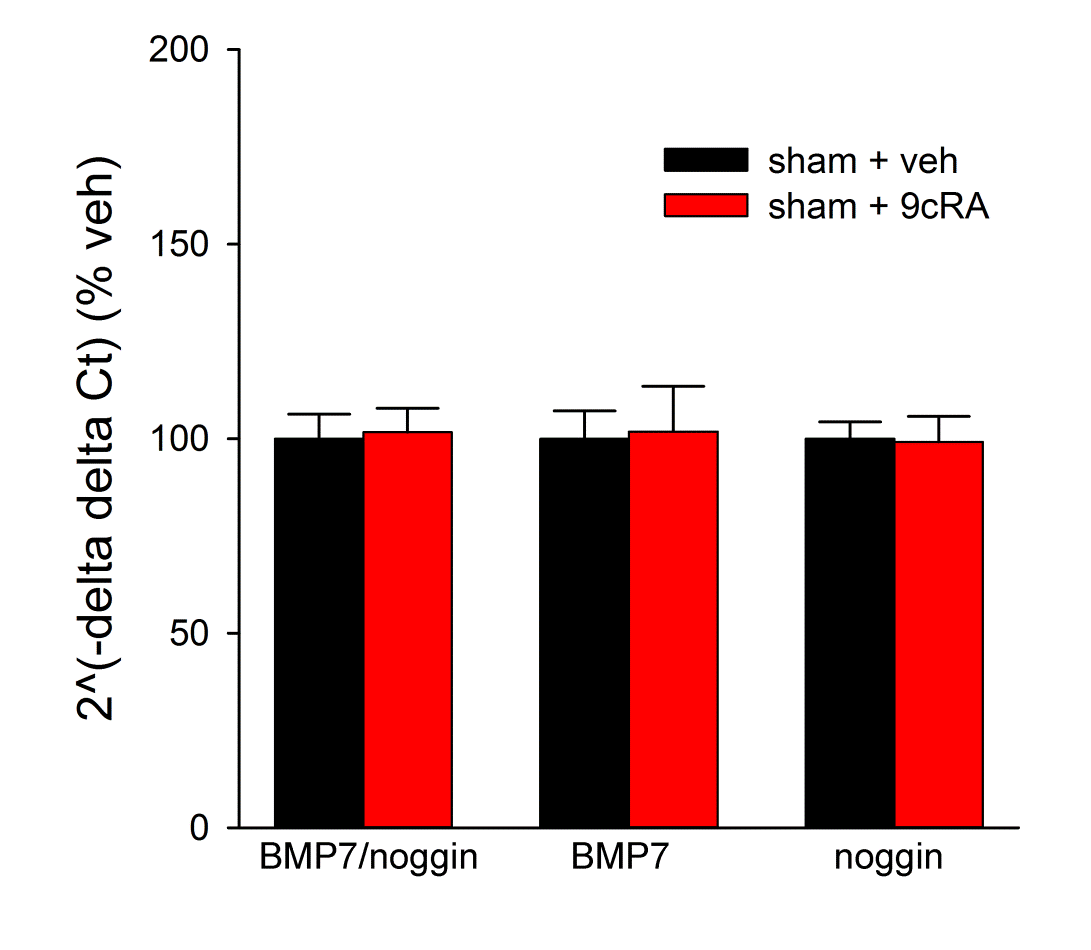


**Supplemental Figure 2: 9cRA did not alter BMP7 expression in the SVZ of animals receiving sham surgery.** (A) Ten rats received intranasal 9cRA or vehicle from days 3 to 6 after dMCAo. SVZ tissues were collected on day 7 to examine the expression of BMP7 and noggin by qRT-PCR as described in Fig 7. Treatment with 9cRA did not alter the expression of BMP7 (p=0.893), noggin (p=0.922) or BMP7/noggin ratio (p=0.852).
